# Sound relief: Restorative Music as a strategy to improve clinician wellbeing

**DOI:** 10.3389/fpsyg.2026.1864986

**Published:** 2026-09-20

**Authors:** N. Salehi, C. Takarabe, M. Walesa, L. Pause, G. Agarwal, B. Bonakdarpour

**Affiliations:** 1 The Ken and Ruth Davy Department of Neurology, Northwestern University Feinberg School of Medicine, Chicago, IL, United States; 2 Department of Wellbeing, Northwestern Medicine, Chicago IL, United States

**Keywords:** clinician wellbeing, music-based intervention, positive emotion, Restorative Music, wellbeing

## Abstract

Over 50% of healthcare providers experience physician burnout, characterized by emotional exhaustion, depersonalization, and an increased risk of medical malpractice. Although burnout is well-documented, fewer studies have examined accessible interventions that may support healthcare professionals during the workday. Studies have associated music-based interventions with reduced stress and increased relaxation. This preliminary study examined whether a brief Restorative Music intervention was associated with short-term changes in emotional state and perceived energy among healthcare professionals. The Restorative Music intervention consisted of 20-min sessions incorporating Clinically Designed Improvisatory Music (CDIM) and Slow Irish Airs selected for calming musical qualities such as slow tempo and simple rhythmic structure. A total of 724 healthcare workers, including physicians, neurology staff, and social workers, accessed the intervention, and 51 participants completed voluntary surveys assessing emotional state, tension, pleasure, likelihood to recommend, and subjective energy. We analyzed pre- and post-intervention energy ratings using a non-parametric Wilcoxon signed-rank test. Participants reported favorable ratings for emotional state and likelihood to recommend. Subjective energy increased from an average of 56% before the intervention to 83% after the intervention; the Wilcoxon signed-rank test confirmed significantly higher post-intervention ratings (W = 13.5, *p* < 0.0001, *r* = 0.85). These preliminary findings support the feasibility and acceptability of Restorative Music as an accessible strategy for promoting short-term clinician wellbeing. Controlled studies using validated wellbeing and burnout measures are needed to determine whether the observed changes are attributable to the music-based intervention.

## Introduction

Burnout is defined as a form of strain that is characterized by sentiments of emotional exhaustion, a lack of personal accomplishment, and depersonalization (Lacy and Chan, 2018). Physicians often experience high levels of burnout because of the inherently stressful nature of medical practice. As a high-risk profession, practicing medicine requires a significant time commitment and carries tremendous responsibility for human lives. Physicians are particularly susceptible to long-term stress, especially those who perform high-risk procedures and face work-life balance challenges (Lacy and Chan, 2018). Occupational stress and burnout are also prevalent among other healthcare professionals, including nurses, trainees, social workers, neurologists and allied healthcare staff, highlighting the need for wellbeing interventions that extend beyond physicians (Marković et al., 2024; Guo et al., 2025). Healthcare burnout not only affects individual healthcare workers’ wellbeing but can also negatively affect patient care; it can double the risk to patient safety and account for 7%–10% of medical malpractice (Shin et al., 2023). With burnout rates affecting over 50% of providers and adversely affecting the medical field, further research is imperative for the efficient maintenance of the healthcare system (Singh et al., 2025). Although current scientific literature often acknowledges the symptoms of physician burnout, more effective interventional strategies are needed to improve the quality and outcomes of burnout reduction (Singh et al., 2025; West et al., 2016). Despite existing programs such as mindfulness strategies and cognitive behavioral training, additional research is needed to determine the efficacy of each program (Razai et al., 2023). Among the possible strategies under investigation, approaches that focus on wellbeing and holistic care have become more prevalent.

Music-based interventions have gained recent attention as one potential strategy and have been shown to reduce stress levels, enhance emotional wellbeing, and induce relaxation (Finnerty et al., 2022). Music may promote wellbeing through its ability to engage multisensory integration, memory, and attention within brain structures involved in cognitive, emotional, and sensorimotor activity (Koelsch, 2009). Music has also shown quantitatively strong effects on reducing heart rate and cortisol levels and decreasing mean arterial pressure (Witte et al., 2022). It can also reduce psychological symptoms, including anxiety, restlessness, and nervousness (Witte et al., 2022). Prior work using Clinically Designed Improvisatory Music (CDIM) in patients admitted to an epilepsy monitoring unit found increased EEG alpha/beta power ratios following exposure to calming music, and these increases were associated with reduced self-reported tension (Bonakdarpour et al., 2023). These preliminary findings provide neurophysiological support for the potential calming effects of CDIM. Together, these findings highlight the physiological and psychological benefits that music may evoke and demonstrate the need for further research on music-based interventions.

Preliminary studies have explored the neurobiological effects of music on burnout. For example, a systematic review determined that music effectively prevented stress, enhanced work engagement, and emotionally supported wellbeing among nurses (Finnerty et al., 2022). Similarly, music-based interventions reduced occupational stress among operating room staff (Kacem et al., 2020). These findings are consistent with improvements in clinicians’ emotional status following a 5-week remote music therapy intervention (Giordano et al., 2020). Music therapy differs from broader music-based interventions because it typically involves the intentional use of music by a trained music therapist to address individualized therapeutic goals, whereas music-based interventions may include structured music listening without formal music therapy (Witte et al., 2022). Although the present intervention was not formal music therapy, we included prior music therapy studies because they provide relevant evidence of how structured musical experiences may influence stress and emotional wellbeing. Music-based approaches have also been effective in reducing stress-related outcomes, such as cortisol levels and anxiety, demonstrating the influence of music on overall wellbeing (Witte et al., 2022).

Despite the favorable results reported in previous literature, researchers should investigate applications of music-based interventions that target a broader spectrum of healthcare workers to increase awareness of these interventions (Chadder, 2019). Although previous studies have focused on specific groups of healthcare workers, they often lack specificity regarding the type of music used. In addition, the broad range of methodologies employed across different populations makes it difficult to standardize the results and draw a well-supported conclusion (Robb et al., 2018).

At our institution, we used two types of music for restoration: CDIM and a selection of song, such as Slow Irish Airs, with calming qualities. These qualities included slow tempi, uncomplicated unsyncopated rhythms, mezzo piano or mezzo forte dynamics, and meandering melodies that do not convey a sense of urgency or teleology and therefore are not heavily cadential. We refer to this combination as “Restorative Music.” This preliminary study examined whether healthcare professionals reported short-term changes in emotional state, tension, pleasure, and perceived energy following exposure to Restorative Music. We collected survey ratings and participant comments to explore the feasibility, acceptability, and perceived short-term effects of Restorative Music. Because we did not directly assess burnout, the study focused on emotional state, tension, pleasure, and subjective energy as indicators of immediate wellbeing rather than as measures of burnout. As an initial exploratory evaluation of a wellness initiative, these findings are intended to inform future controlled studies of music-based interventions for healthcare professionals.

## Materials and methods

### Participants

This study was initiated during the COVID-19 pandemic and continued beyond the pandemic period. Participants included physicians and neurology residents at Northwestern Medicine who accessed prerecorded Restorative Music through the Recuperation Room, as well as psychiatric social workers who participated in separate in-person live music sessions. The delivery format differed because we adapted the intervention to the practical constraints and available resources within each participating group. We used voluntary sampling to obtain perspectives from multiple healthcare professions and to explore the perceived effects of Restorative Music across these groups. Participants completed the surveys on their own time to allow sufficient reflection and minimize potential subconscious influence from the researchers. This study did not require IRB oversight or ethical approval because data collection involved fully anonymous surveys and recorded no personal or health information. Participants indicated informed consent by voluntarily and anonymously completing the surveys after receiving information about the study’s purpose.

### Music-based intervention

The Restorative Music intervention comprised approximately 20-min sessions incorporating CDIM and Slow Irish Airs. Physicians and neurology participants received prerecorded Restorative Music selections, whereas a Certified Music Practitioner (CMP) performed live music for social workers. A CMP is a musician trained through the Music for Healing and Transition Program (MHTP) to provide acoustic music in healthcare settings for therapeutic purposes and to address patients’ immediate needs (Music for Healing and Transition Program, n.d.). We selected a 20-min duration for both the prerecorded and live Restorative Music sessions to provide a meaningful period of restorative listening while remaining feasible within the time constraints of a healthcare workday. The intervention included seven segments: four CDIM improvisation pieces (Improvisations 1–4) and three Slow Irish Airs (Slow Irish Air 1 and 2, and Lament for the Fox). One piece (Geaftai Bhaile Bui) combined improvisational elements with a Slow Irish Air. We chose these selections for their calming musical qualities, including slow tempo, minimal rhythmic complexity, and greater tonal stability (Bernardi et al., 2006; Zhang et al., 2022). For psychiatric social workers, a CMP (author CT) performed the CDIM selections as live on the viola, whereas we presented the Slow Irish Air selections as prerecorded music. Physicians and neurology residents accessed prerecorded versions of both the CDIM and Slow Irish Air selections through Northwestern Memorial Hospital’s Recuperation Room, where the recordings were available 24 h a day through a dedicated website.^1^

The CDIM design aimed to evoke feelings of safety and support parasympathetic activity through specific musical characteristics. CDIM uses a limited pitch range, a slow tempo of approximately 50–70 beats per minute, simple unsyncopated rhythms, meandering melodies, and gradual pitch transitions. We selected the viola because its pitch range overlaps with the human vocal range. Furthermore, Polyvagal Theory informed the design of CDIM. This theory proposes that perceived cues of safety can promote autonomic regulation through vagal pathways associated with parasympathetic activity and social engagement (Porges, 2011). We therefore selected CDIM’s musical features to create a predictable, low-arousal auditory environment intended to support relaxation. Prior work using CDIM has reported improved self-reported emotional state and reduced tension, accompanied by increased EEG alpha/beta power ratios associated with relaxation (Bonakdarpour et al., 2023).

Irish airs are slow, expressive melodies that often originate from traditional old-style singing. Unlike dance tunes, airs do not follow a strict rhythm and instead follow the natural phrasing of the song lyrics on which they are based. Some Irish airs use Dorian or Mixolydian modes; Dorian has a minor quality with a raised sixth scale degree, whereas Mixolydian has a major quality with a lowered seventh. The present intervention used unaccompanied Slow Irish Air selections recommended by the MHTP (Golden, 1979; Music for Healing and Transition Program, n.d.).

A linked survey asked four primary questions, including questions about the likelihood of recommending Restorative Music, the likelihood of recommending the Recuperation Room, and pre- and post-intervention energy levels. We also distributed Restorative Music within the Neurology Department, including to residents, faculty, and psychiatric social workers. After the 20-min Restorative Music session, we conducted unstructured interviews to assess participants’ perceived changes in emotional state. We selected unstructured interviews to allow participants to describe their experiences in their own words without restricting responses to predetermined categories and to provide contextual descriptive feedback alongside the quantitative survey ratings. Participants in both the Neurology Department and social worker groups rated their experiences on a scale of 1–10 for changes in positive emotions, decreased tension, pleasure, overall energy, pre/post energy level, and likelihood of recommending Restorative Music. Participants submitted survey responses via a QR code or physical copies; neither format listed participant names. We entered the responses into a combined spreadsheet and collected them from October 2022 through August 2025. We averaged the raw Likert-scale survey data and converted them into visual graphs. We used method triangulation by combining surveys and interviews with thick descriptions that included participant categorization, setting, and interview duration.

### Statistical analysis

We compared pre- and post-intervention subjective energy ratings using the nonparametric Wilcoxon signed-rank test among participants with complete paired responses. Statistical significance was defined at α = 0.05. We conducted the analysis using the StatsKingdom Wilcoxon Signed-Rank Test Calculator (StatsKingdom, n.d.).

## Results

A total of 724 individuals participated in the Restorative Music session. This included 493 NM healthcare providers, 60 Neurology Department faculty members (residents and students), and 171 social workers. We received voluntary responses from 17 NM physicians, 21 Neurology Department staff members, and 13 social workers (Table 1). This represented an overall survey response rate of 7.0% (51 of 724 participants).

**TABLE 1:** Restorative Music participants.

Group	Participated	Responded
NM physicians	493	17
Neurology dept.	60	21
Social workers	171	13
Total	724	51

The number of attendees and number of responses are demonstrated.

The survey included eight questions regarding the Recuperation Room and Restorative Music (Table 2).

**TABLE 2:** Questions from the recuperation room and Restorative Music survey.

Question number	Question	Response format
1	The Restorative Music session improved my mental and emotional state.	Likert scale
2	The Restorative Music session improved any feelings of tension.	Likert scale
3	The Restorative Music session improved my ability to experience pleasure and contentment.	Likert scale
4	The Restorative Music session improved my energy level.	Likert scale
5	How full was your tank today before the session?	Likert scale
6	How full is your tank now after the music session?	Likert scale
7	Would you like Restorative Music to continue regularly?	Likert scale
8	Would you like the recuperation room to continue regularly?	Likert scale

As shown in Figure 1, participants reported positive effects on emotion at 84%, decreased tension at 86%, pleasure levels at 84%, and overall energy improvement at 86%. Moreover, participants rated their likelihood of recommending Restorative Music at 95% and the Recuperation Room at 97%.

**FIGURE 1:**
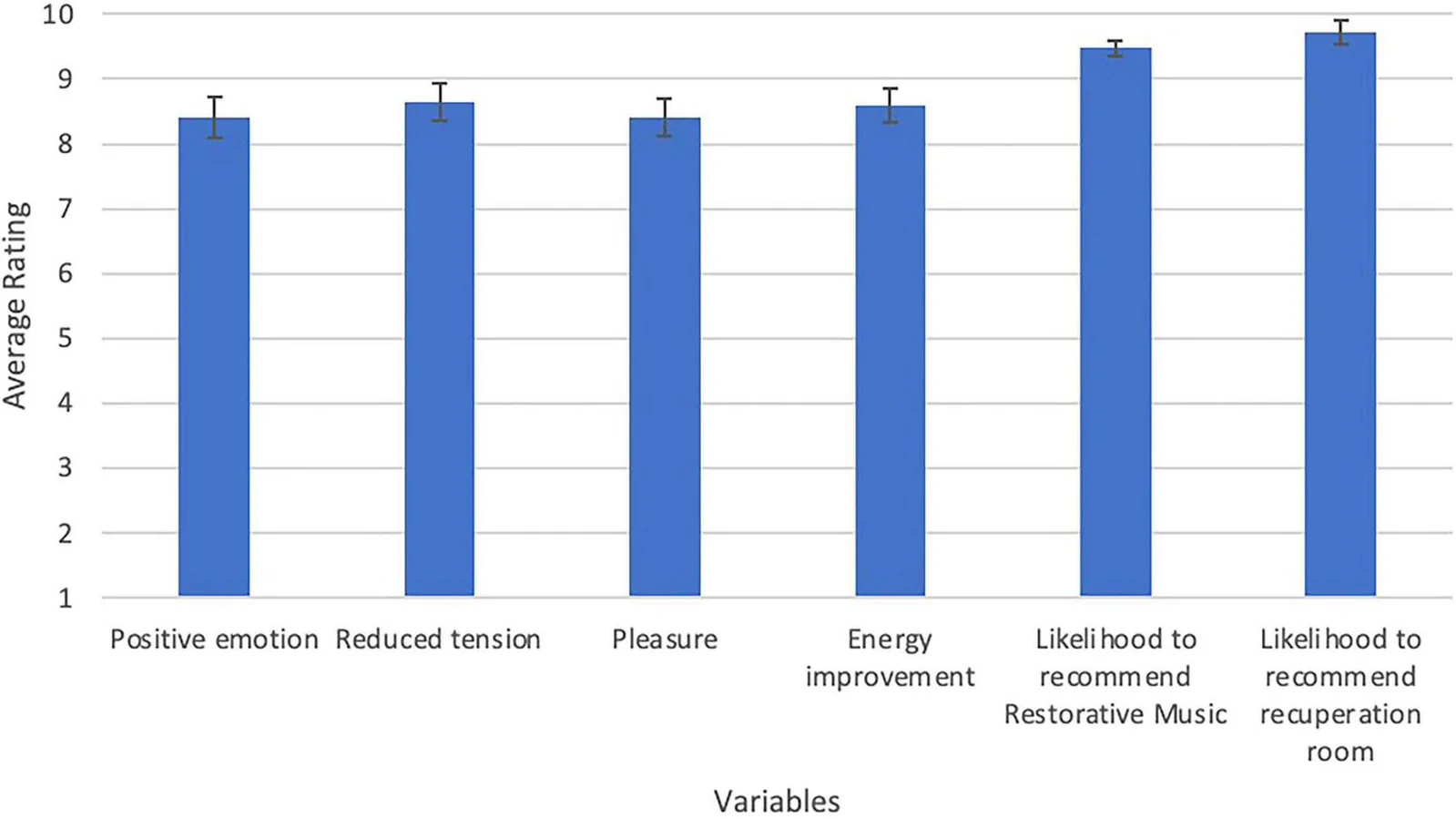
Restorative Music session averages reported from Northwestern Medicines physicians (neurology and non-neurology), and community social workers on the Likert scale (1–10) measuring positive emotion, decreased tension, pleasure, energy level, and likelihood to recommend Restorative Music, likelihood to recommend CDIM, and likelihood to recommend the recuperation room.

Subjective energy ratings increased from an average of 56% before the intervention to 83% after the intervention (Figure 2). The Wilcoxon signed-rank test showed significantly higher post-intervention energy ratings than pre-intervention ratings (W = 13.5, *r* = 0.85, *p* < 0.0001). Participants provided positive feedback on the survey, with comments such as “very soothing space. Glad it’s here,” “absolutely great experience to take some time out during our busy schedules and appreciate the beautiful music being performed lived,” and “fantastic way to improve mental and physical wellbeing!”

**FIGURE 2:**
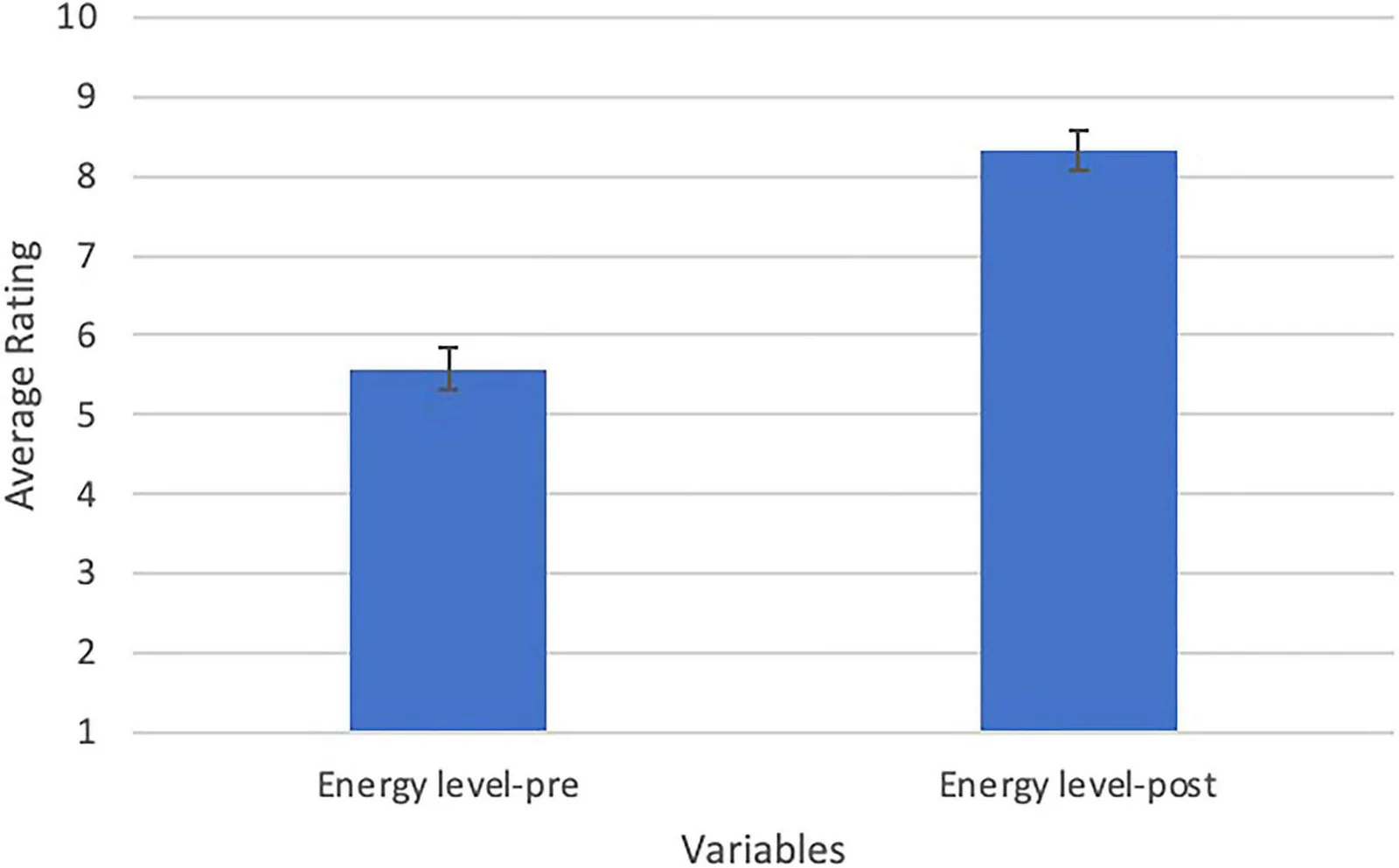
Subjective energy level ratings before and after Restorative Music sessions across all participants.

## Discussion

In this study, we investigated the effects of Restorative Music among healthcare providers, including neurologists, neurology trainees, other physicians, and psychiatric social workers. After listening to 20 min of Restorative Music, participants reported positive effects on emotional valence, decreased tension, and a high likelihood of recommending the intervention. They also reported notably improved subjective energy levels.

These findings suggest that participants found Restorative Music acceptable and reported favorable emotional states and subjective energy immediately following the sessions. However, because the study did not include a control condition, we cannot attribute the observed changes specifically to the music-based intervention. The findings are consistent with previous studies reporting potential benefits of music-based interventions for stress and wellbeing among healthcare workers (Finnerty et al., 2022; Witte et al., 2022; Kacem et al., 2020; Giordano et al., 2020).

Burnout can affect emotions (e.g., increased tension and decreased pleasure) and energy levels. Previous literature has found that positive-emotion skills can help prevent burnout, whereas negative emotions increase with burnout symptoms (Cheung et al., 2020; Szczygiel and Mikolajczak, 2018). Negative emotions, burnout, and decreased energy are closely interrelated. Negative emotions can contribute to burnout, burnout can intensify negative emotional experiences, and both factors are associated with reductions in energy levels. This cyclical relationship may perpetuate symptoms and contribute to ongoing emotional and physical depletion. The present study assessed short-term emotional state, tension, pleasure, and perceived energy; these variables may be relevant to clinician wellbeing but should not be interpreted as direct measures of burnout. In our study, participants reported positive emotion and reduced tension, reflected in mean Likert scores of 84% and 86%, respectively. Burnout is further characterized by a sense of energy depletion, reduced drive, and emotional fatigue (Khammissa et al., 2022). Participants reported increased energy, with Likert scores rising from 56% to 83%. Despite these favorable findings, changes in emotion, tension, pleasure, and energy should be interpreted as short-term indicators of wellbeing that may be relevant to burnout-related experiences rather than as direct measures of burnout itself.

Moreover, music plays a role in metabolic recovery from stress by decreasing hypothalamic-pituitary-adrenal (HPA) axis stimulation while influencing sympathetic nervous system (SNS) and immune system activity (Yamasaki et al., 2012). Reductions in heart rate and blood pressure have demonstrated decreased sympathetic activity (Lata and Kourtesis, 2021).

Previous research has reported that music-based interventions may reduce burnout among nurses, decrease occupational stress among operating-room staff, and improve clinician wellbeing (Finnerty et al., 2022; Kacem et al., 2020; Giordano et al., 2020). The present study extends this literature by examining a structured calming-music intervention across several groups of healthcare professionals. Although previous studies primarily focused on a subsection of healthcare workers, this study successfully applied Restorative Music across multiple groups of healthcare workers.

## Limitations

Despite these findings, several limitations must be acknowledged. First, the study relied on subjective, self-reported Likert-scale ratings, and recall bias, social desirability, and participant expectations may have influenced these ratings. In addition, we did not directly measure burnout using a validated instrument. Although emotional state, tension, pleasure, and energy may relate to occupational wellbeing, these measures cannot establish a reduction in burnout. Future studies should include validated measures such as the Maslach Burnout Inventory or Copenhagen Burnout Inventory and should distinguish immediate emotional responses from longer-term occupational burnout (Li et al., 2024).

Future studies could also incorporate more quantitative measures of stress, such as physiological indicators [heart rate, blood pressure, cutaneous response, hormone levels (cortisol, oxytocin), and immunologic markers]. In addition, our study lacked a control group for comparison. Therefore, the observed changes may reflect the effects of taking a break, resting in a calming environment, or participant expectations rather than the music-based intervention alone.

We originally implemented this intervention during the COVID-19 pandemic as a supportive wellness initiative rather than a controlled research study. Pandemic-related operational limitations, together with the goal of providing a low-burden restorative experience for healthcare workers, limited the feasibility of incorporating a control condition during the initial implementation. Future studies should include a control condition in which participants spend an equivalent 20-min period resting quietly without music. A controlled comparison would help determine whether the observed improvements are attributable specifically to Restorative Music rather than to non-specific effects such as taking a break, resting in a calming environment, or participant expectations. Moreover, the overall response rate was low, limiting the generalizability of our findings and introducing the possibility of selection bias because participants with more favorable experiences may have been more likely to respond. However, we considered mandatory survey completion inconsistent with the voluntary and restorative nature of the intervention. Moreover, because we developed the intervention primarily as a wellness initiative during the COVID-19 pandemic, we intentionally kept data collection minimally burdensome and did not incorporate objective physiological measures into the initial implementation.

## Conclusion

Restorative Music was feasible to implement, and respondents viewed it favorably. Participants reported positive emotional states, reduced tension, pleasure, and higher subjective energy following the sessions. Because the study lacked a control group and did not directly assess burnout, these findings should be interpreted as preliminary evidence of acceptability and short-term perceived wellbeing rather than evidence of clinical effectiveness or burnout reduction. Future controlled studies should include larger samples, validated burnout and wellbeing instruments, physiological measures, control or comparison groups, and longer-term follow-up. These preliminary findings support further controlled evaluation of Restorative Music as a feasible and acceptable strategy for promoting short-term clinician wellbeing.

## Data Availability

The raw data supporting the conclusions of this article will be made available by the authors, without undue reservation.

## Footnotes

1
https://www.bornacogneurology.com/music-and-medicine-program/programs/restorative-music
